# Delayed versus immediate surgical intervention for perforated appendicitis

**DOI:** 10.6026/973206300211612

**Published:** 2025-06-30

**Authors:** J. Naveen Bose, Rhea Khamitkar, Emilda Ambrose, Kaushik Nattamai Rameshbabu, Priyanka Perumal, Toufique Khan, Keerthika Muniasamy, Shoraf P.

**Affiliations:** 1 Department of Emergency Medicine, Madurai Medical College, Panagal Rd, Alwarpuram, Madurai, Tamil Nadu, India; 2 Department of Internal Medicine, Sparsh Hospital Bengaluru, Karnataka, India; 3 Department of Emergency Medicine, St. Joseph's Hospital, Dharmagiri, Kothamangalam, Ernakulam, Kerala, India; 4 Department of Surgery, Madras Medical College, Chennai, Tamil Nadu, India; 5 Department of General Surgery, SRM Medical College Hospital and Research Centre, Chengalpattu, Tamil Nadu, India; 6 Department of Acute Medicine and Emergency, Labaid Specialized Hospital and Diagnostic Center, Dhaka 1205, Bangladesh; 7 Department of General Surgery, Madras Medical College, Chennai, Tamil Nadu, India; 8 Department of Community Medicine, Madha Medical College and Research Institute, Kovur, Chennai, Tamil Nadu, India

**Keywords:** Appendicitis, perforation, surgery, immediate intervention, delayed intervention, postoperative complications, emergency medicine

## Abstract

Perforating appendicitis is a critical condition that needs urgent surgery. However, there is controversy exists regarding early versus delayed surgery. Hence, we assessed 100 patients with immediate surgery in 50 cases and delayed intervention in the remaining 50 cases. The patients were followed up for one year to determine postoperative complications, hospital stay, and recovery. Results indicated that early surgery decreased complications and hospital stay very significantly. These results underscore the significance of prompt surgical intervention in enhancing outcomes and lowering healthcare costs.

## Background:

A serious complication of acute appendicitis is perforated appendix. This condition arises because a perforated inflamed appendix leaks infectious material into the peritoneal cavity. Such disease would pose a high risk for fatal complications such as sepsis and peritonitis, hence requiring urgent surgery [1]. Although appendectomy remains the treatment of choice for perforations of the appendix, decisions in emergency medicine and surgery remain debatable in whether intervention should be delayed or done immediately. After the diagnosis has been made, an appendectomy is performed as immediate surgical intervention [2]. It is usually carried out a few hours after admission to the hospital. The main aim is to further delay the progression of infection as well as all its consequences. The patient first undergoes supportive care, fluid resuscitation and antibiotics within delayed intervention [3]. The surgery is only done after the improvement of the condition of the patient. A patient whose condition is marred by serious inflammation, abscess formation, or haemodynamic instability is most likely to benefit more from the delay of surgery although immediate surgery is associated with lower infection-related morbidity [4]. Intervening immediately or delaying intervention depends on various parameters including the clinical presentation of the patient, comorbidities and the available medical resources [5]. The mixed results of studies investigating the outcomes of these therapies show that although some argue for an immediate attempt to minimize complications postoperatively, others recommend a delayed approach, which is akin to proper preoperative care [6]. Therefore, it is of interest to compare the outcomes of emergency versus delayed surgical surgery for perforated appendicitis in a tertiary care setting. The parameters considered include postoperative complications, length of stay in the hospital, recovery period and use of health resources.

## Materials and Methods:

This prospective comparative study evaluates the immediate compared to the delayed surgical intervention in a tertiary care hospital setting for perforated appendicitis for a 12-month period. Of the 100 patients recruited into this study, there were two arms of treatment: immediate surgery six hours after the diagnosis and delayed surgery conducted 24 to 72 hours after first stabilization. Inclusion criteria included at least eighteen years of age, radiologic and clinical proof of perforated appendicitis and consent to participate in the study. Exclusion criteria included refusal of therapy, serious comorbidity rendering surgery inadvisable, or advanced sepsis. Baseline results of tests, clinical presentations and demographic details were obtained on admission. In the immediate intervention group, the patients were operated on after receiving standard preparation preoperatively, fluid resuscitation and intravenous antibiotics. In the delayed intervention group, stabilization was first achieved with broad-spectrum antibiotics, analgesics and fluid management to control the infection and inflammation before surgery. All surgeries were performed using either open or laparoscopic techniques based on the surgeon's discretion and clinical condition of the patient. Outcomes of wound infections, intra-abdominal abscesses and sepsis post-surgery were evaluated. Other parameters were the length of stay in the hospital, days taken to recover and readmission or reoperation rate. Data was analyzed with appropriate statistical methods for comparison of outcomes between both arms. Institutional approval was sought through institutional review board and informed consent was obtained from each participant.

## Results:

Table 1 (see PDF) Summarizes the baseline characteristics of the patients studied. Both cohorts were similar regarding age, gender and comorbid conditions, which thus balanced the comparison. Table 2 (see PDF) Compares incidence of postoperative complications between two groups. Synchronous surgery presented a lower absolute rate of total complications compared with delayed surgery. Table 3 (see PDF) highlights the mean number of days a patient stayed in the hospital by group. Early surgery significantly shortened the length of stay compared to delayed intervention. Table 4 (see PDF) details the recovery time to normal activity. Patients in the immediate surgery group demonstrated faster recovery compared to those in the delayed surgery group. Table 5 (see PDF) summarizes readmission and reoperation rates. The immediate surgery group had fewer readmissions and reoperations compared to the delayed group. Table 6 (see PDF) illustrates healthcare resource utilization, including total costs incurred per patient. Immediate surgery resulted in significantly lower costs compared to delayed intervention. The study showed significant differences between immediate and delayed surgical intervention for perforated appendicitis across several parameters. Baseline characteristics (Table 1 - see PDF) were comparable in terms of age, gender distribution and comorbidities between groups, thus ensuring a balanced comparison. Postoperative complications (Table 2 - see PDF) were significantly lower in the immediate surgery group, with a total complication rate of 32% compared to 64% in the delayed group. The length of stay in the hospital was shorter among the immediate surgery group; its average length was 6.5 days against 9.2 days of the delayed surgery group (Table 3 - see PDF). Recovery to normal activity (Table 4 - see PDF) was shorter in the case of immediate surgery compared to delayed group, whose recovery time to normal activity averaged at 19.4 days as against 14.8 days. Readmission and reoperation rates (Table 5 - see PDF) were also less for the immediate group, readmitted at 4% and required reoperations at 2% compared to the delayed group of 14% and 10%, respectively. Healthcare resource utilization (Table 6 - see PDF) reveals that immediate surgery was costlier, averaging around INR 32,000 per patient compared to the delayed group which averaged around INR 42,000 per patient. These findings put emphasis on clinical and economic merits of immediate surgery in perforated appendicitis.

## Discussion:

Thus, in perforated appendicitis, the results of this study show that there is a clear advantage of immediate surgery over delayed intervention [7]. Patients who underwent urgent surgery experienced better clinical outcomes, such as fewer postoperative complications, shorter hospital stay and faster recovery. As supported by earlier studies, the results indicate that patients should be treated early before surgery in order to contain the spreading infection and its implications [8]. Although it is at a general complication rate of 32%, the emergency surgery group had a lower complication post-surgery compared to the delayed surgery in which it is as high as 64%. This huge difference points to potential dangers in delaying surgery, since increased exposure to peritoneal contamination may worsen the disease [9]. In addition, while the mean hospital stay for the immediate group was 6.5 days, that of the delayed group was 9.2 days. This lessened duration of hospital stay both enhances the patients' outcomes and reduces the consumption of healthcare resources, as determined by the reduced total cost incurred in the urgent surgery group [10]. Patients who were operated promptly recovered to their regular activities much faster, which can be partly due to lesser morbidity in postoperative and faster resolution of infection [11]. The early group of patients resumed their usual activities at 14.8 days, whereas the delayed surgery group resumed at an average of 19.4 days. The outcome suggests that the delays in surgical care should be minimized to achieve early functional recovery. The benefit of early surgery is also enhanced by the readmission and reoperation rates [12]. These also translated into far higher readmission and reoperation rates, hinting at associated risks and inefficiencies in prolonged preoperative care. As such, intervention via timely surgical therapy is not only safer but more cost-effective than interventions and the related problems. The cost-effectiveness of urgent surgery was higher. It offered a mean total cost of INR 32,000 per patient as compared to INR 42,000 when intervention is delayed. This can be ascribed to short hospital stays, fewer problems and fewer surgeries or additional procedures [13]. The cost-effectiveness of using urgent surgery to treat ruptured appendicitis also supports its need. Though the study is incredibly informative, the limits of it should be well recognized. As the sample size is restricted to only 100 patients, the findings are not as generalizable as they could have been. Long-term results and quality-of-life assessments, which would usually be paramount considerations in surgical techniques, are also excluded in this study [14]. Further research with larger sample sizes and longer follow-up periods is necessary to confirm the outcomes obtained here and to investigate other outcome indicators. This study proves that early surgical surgery for perforated appendicitis has a definite advantage since it improves recovery, saves health costs and minimizes complications. Therefore, our data support the traditional practice of treating this situation urgently with immediate surgery, particularly in limited resource settings [15].

## Conclusion:

The immediate surgical intervention for perforated appendicitis causes fewer complications, a reduced stay in the hospital and a faster recovery. It is also more economical since it makes use of less medical facility while ensuring better results for the patients. These facts, therefore, support the new idea of treating perforated appendicitis as an emergency intervention.

## References

[1] Yardeni D (2004). J Pediatr Surg..

[2] Koger K.E (1996). Am Surg..

[3] Siribumrungwong B (2018). Ann Surg..

[4] Saluja S (2018). J Pediatr Surg..

[5] Abdulhamid A.K (2018). Ann Med Surg (Lond)..

[6] Weber T.R (2003). Am J Surg..

[7] Roach J.P (2007). Am J Surg..

[8] Pokorny W.J (1991). Surg Gynecol Obstet..

[9] Patel S.V (2024). Ann Surg..

[10] Zhou S (2024). Cochrane Database Syst Rev..

[11] Javed H (2023). Cureus..

[12] Boettcher M (2017). Clin Pediatr (Phila)..

[13] Balogun O.S (2019). Ann Afr Med..

[14] Cui C (2020). Asian J Surg..

[15] Villalón U.H (2020). Rev Chil Pediatr..

